# The Blunt Liver and Spleen Trauma (BLAST) audit: national survey and prospective audit of children with blunt liver and spleen trauma in major trauma centres

**DOI:** 10.1007/s00068-022-01990-3

**Published:** 2022-06-21

**Authors:** Paediatric Surgery Trainee Research Network, R. Harwood, G. Bethell, M. P. Eastwood, S. Hotonu, B. Allin, T. Boam, C. M. Rees, N. J. Hall, H. Rhodes, T. Ampirska, F. Arthur, J. Billington, G. Bough, O. Burdall, K. Burnand, S. Chhabra, C. Driver, J. Ducey, N. Engall, E. Folaranmi, D. Gracie, K. Ford, C. Fox, P. Green, S. Green, W. Jawaid, M. John, C. Koh, C. Lam, S. Lewis, R. Lindley, D. Macafee, I. Marks, L. McNickle, B. J. O’Sullivan, R. Peeraully, L. Phillips, A. Rooney, H. Thompson, L. Tullie, S. Vecchione, A. Tyraskis, B. Nezafat Maldonado, M. Pissaridou, N. Sanchez-Thompson, L. Morris, M. John, A. Godse, P. Farrelly, P. Cullis, M. McHoney, D. Colvin

**Affiliations:** Collaborative National Research Group, Alder Hey in the Park, East Prescot Road, Liverpool, Merseyside UK

**Keywords:** Trauma, Pediatric, Blunt injury, Liver Spleen

## Abstract

**Purpose:**

To compare the reported and observed management of UK children with blunt liver or spleen injury (BLSI) to the American Pediatric Surgical Association (APSA) 2019 BLSI guidance.

**Methods:**

UK Paediatric Major Trauma Centres (pMTCs) undertook 1 year of prospective data collection on children admitted to or discussed with those centres with BLSI and an online questionnaire was distributed to all consultants who care for children with BLSI in those centres.

**Results:**

All 21/21 (100%) pMTCs participated; 131 patients were included and 100/152 (65%) consultants responded to the survey. ICU care was reported and observed to be primarily determined using haemodynamic status or concomitant injuries rather than injury grade, in accordance with APSA guidance. Bed rest was reported to be determined by grade of injury by 63% of survey respondents and observed in a similar proportion of patients. Contrary to APSA guidance, follow-up radiological assessment of the injured spleen or liver was undertaken in 44% of patients before discharge and 32% after discharge, the majority of whom were asymptomatic.

**Conclusions:**

UK management of BLSI differs from many aspects of APSA guidance. A shift towards using clinical features to determine ICU admission and readiness for discharge is demonstrated, in line with a strong evidence base. However, routine bed rest and re-imaging after BLSI is common, contrary to APSA guidance. This disparity may exist due to concern that evidence around the incidence, presentation and natural history of complications after conservatively managed BLSI, particularly bleeding from pseudoaneurysms, is weak.

**Supplementary Information:**

The online version contains supplementary material available at 10.1007/s00068-022-01990-3.

## Introduction

Every year in the United Kingdom (UK) approximately 130 children experience blunt abdominal trauma with solid organ injury [1]. A significant proportion of these children require a hospital stay and potentially surgery [2]. There has been a shift in the management of blunt liver and spleen injuries (BLSI) over the past 40 years [3–7], primarily after the release of the American Pediatric Surgery Association (APSA) guidelines in 2000 [8]. This guidance highlighted that the majority of paediatric BLSI could be managed conservatively, and recommended a period of observation based on grade of injury + 1 day, with no requirement for follow-up imaging [8, 9].

Since then, large collaboratives in the United States of America have developed management protocols based primarily on haemodynamic status rather than the grade of injury [10, 11]. However, late complications of trauma including pseudoaneurysm formation and delayed bleeding have been reported as the majority of children are now managed conservatively [12]. There appears to be a higher risk of vascular abnormality (pseudoaneurysm) in higher grade injury but the evidence to support recommendations for routine imaging or intervention are weak [5, 12].

Recently APSA published a systematic review considering the length of stay and level of care, restriction of activity, the role of interventional radiology (IR) and follow-up imaging studies for children managed non-operatively after BLSI [5]. Subsequently new APSA 2019 guidelines for the management of BLSI have been released [13]. Understanding the application of these ‘best-practice’ guidelines by surgeons in the UK is important, particularly as there are no UK or European guidelines and as management varies between specialist and non-specialist centres [14].

The evidence supporting the APSA guidance on acute assessment and management immediately after BLSI is strong [5, 10]. If UK surgeons routinely keep children in hospital for longer than is necessary, or have a lower threshold for intensive care admission, then applying the APSA guidance may enable early discharge and appropriate allocation of resources [10]. Additionally, the variability in management and outcomes that has previously been demonstrated within the UK [14] may be improved by employing evidence based pathways. However, questions remain about the utilisation of additional imaging to identify complications such as pseudoaneurysm after BLSI and the timing of return to activity and contact sport after injury. Assessment of UK clinicians’ approach and variation from APSA guidance is therefore important since it demonstrates what specialist UK surgeons feel is a safe approach and may help to identify evidence gaps and areas of equipoise which could inform future research into these more contentious areas.

The aim of this study is to describe the current approach to paediatric BLSI management by UK paediatric trauma specialists and compare this to the APSA 2019 guidance.

## Materials and methods

The STROBE and CHERRIES checklist were used to describe the relevant methodology for the e-survey and the observational, prospective audit (Supplementary Information) [15, 16].

### Survey design

An online survey was developed in REDCap [17, 18] (Research Electronic Data Capture, Vanderbilt University), comprising questions based on the APSA 2019 guidance to elucidate the approach to BLSI. The questions focussed on the following aspects of care for BLSI:

1. Criteria used for admission to the high dependency and intensive care units
2. Duration of bed rest
3. Criteria for discharge
4. Guidance given to patients about return to sport
5. Post-injury imaging (after initial computed tomography (CT) scan)—timing and modality

Respondents were given multiple choice options for each question with the opportunity to add free text if the choices did not reflect their approach. The questionnaire can be found in the Supplementary Information pages 2–3.

#### Survey pre-testing

The survey was internally validated by a selection of consultant paediatric surgeons prior to production in its final form.

#### Recruitment and administration of the survey

A collaborator was identified in each pMTC in the UK giving a total number of 21 centres. Collaborators distributed the link to the questionnaire to all consultant surgeons in their centre who care for children under 18 years of age with BLSI and informed the study team of the maximum number of responses to expect. The collaborators informed the respondents about the reason for the study, who was undertaking it and that the responses were anonymised. The survey was open for 6 weeks in November and December 2019. Reminders to complete the questionnaire were sent to consultants during this period.

Responses were excluded if the questionnaire was only partially completed to avoid duplication of responses, or if the respondent only cared for people aged 18 years and above.

### Prospective data collection

Children under 18 years of age with radiologically confirmed BLSI between January 2020 and January 2021 were identified prospectively within each pMTC. Observational data were collected from the time of admission to 6 months after injury using patient notes, radiological imaging and laboratory results. Telephone consultations between peripheral hospitals and pMTCs were also captured for patients who were not transferred to a pMTC. Grade of injury was determined using the American Association for the Surgery of Trauma (AAST) grading[19] and was undertaken by radiologists in each unit. Anonymised data were entered into an online Redcap database [17, 18] (Research Electronic Data Capture, Vanderbilt University) held securely by the University of Southampton.

#### Ethical approval

Respondents to the survey consented to participate and all responses were held securely. The project was registered at each participating centre as an audit of practice compared to APSA guidance. Prospective data collection was performed by members of the direct care team. No individual patient identifiable data were held in the Redcap database. Ethical approval was not required according to the Health Research Authority Decision Tool [20].

### Data analysis

Descriptive statistics are used to compare survey responses and patient data to APSA guidance. Missing data are reported where relevant and follow-up data are taken as recorded by the local centre. Mann–Whitney *U* test is used to compare non-parametric data and statistical significance is taken as *p* < 0.05.

## Results

One-hundred and thirty one children with BLSI were reported to the audit during the year of data collection. Road traffic injuries were the most common cause of injury (41, 31%), followed by cycling injuries (37, 28%), fall from a height (22, 17%) and horse-riding injuries (10, 8%). One patient did not survive their multiple injuries and died before leaving the emergency department. 52 (40%) children had a liver injury (18 (14%) isolated liver injury), 66 (50%) had a splenic injury (34 (26%) isolated splenic injury) and 13 (10%) had both liver and spleen injury (without additional concomitant injuries in 2 (2%)). The most common sites of concomitant injuries were the lungs (41, 31%), chest wall (27, 20%) and the kidney (23, 17%) (Table 1). For all patients, the median grade of splenic injury was III (IQR II–IV) and of liver injury was III (IQR II–III) The Injury Severity Score (ISS) was recorded in 59 patients who had a median ISS of 16 (IQR 7.5–24, range 4–57). Four children, all with grade IV and V injury and haemodynamic instability, underwent splenic vessel embolization and all avoided subsequent laparotomy. Seven children had an early laparotomy: 2 had a splenectomy, 2 had packing and preservation of the spleen and oversewing of a gastric perforation, 2 had packing of the liver and one had repair of an aortic bleed. Two patients had a delayed bile leak.

**Table 1:** Concomitant injuries sustained at the same time as liver or spleen injury

Additional organs injured	77/131 (59%)
Lungs	41 (31%)
Chest Wall	27 (20%)
Kidney	23 (18%)
Other	21 (16%)
Brain	12 (9%)
Pelvis	6 (5%)
Spine	6 (5%)
Bowel	6 (5%)
Heart	< 5 (< 4%)
Aorta	< 5(< 4%)
Pancreas	< 5 (< 4%)
Femur	< 5 (< 4%)
Humerus	< 5 (< 4%)
Bladder	0

### Survey respondents

One hundred and fifty two surgeons were invited to participate and 100 completed surveys were returned (66% response rate). All 21 pMTCs/specialist units were represented in the responses, including the 3 specialist UK liver units. Trauma was reported as a subspecialist interest by 27/100 (27%) and 4/100 (4%) reported trauma as their only subspecialty area. 7/100 (7%) respondents were hepatobiliary specialists. One respondent only reviews patients in the acute phase of the admission and therefore was not included in follow-up responses.

**APSA guidance: admission to ICU is indicated if there are abnormal vital signs after initial resuscitation.**

Thirty four (26%) children were admitted to the Intensive Care Unit (ICU) on the first day of admission, 21 (62%) of whom had required resuscitation prior to admission (fluid bolus or blood products). Children admitted to ICU requiring resuscitation did not have a significantly different grade of injury to those not requiring resuscitation (resuscitation given: 3 (IQR 2–4) vs no resuscitation given: 3.5 (IQR 2.8–4.3), p 0.53). 16/21 (76%) of those admitted to ICU and given fluid resuscitation had injury of at least one other organ and 10/13 (77%) not given resuscitation had injury to another organ. 3 patients who had neither multiple injuries nor required resuscitation were admitted to ICU, all of whom had a grade III injury or above.

### Survey respondents

45/100 (45%) of survey respondents report placing children on ICU based on haemodynamic features of instability without using injury grade as an indication. 10/100 (10%) use grade in combination with haemodynamic status and the majority of these respondents also report using concomitant injuries as an additional factor in determining whether ICU admission is indicated.

Grade of injury was reported as the sole determinant of ICU admission for 15/100 (15%) of respondents. For those who admit to ICU based on grade, 2/15 (13%) do so for Grade III and above, 8/15 (53%) do so for Grade IV and above and 5/15 (33%) do so for Grade V.

The remaining respondents did not give a description of the indications which they use for admission.

**APSA guidance: Bedrest on ICU until all vitals are normal. No restriction of activity on the ward.**

**Fig. 1:**
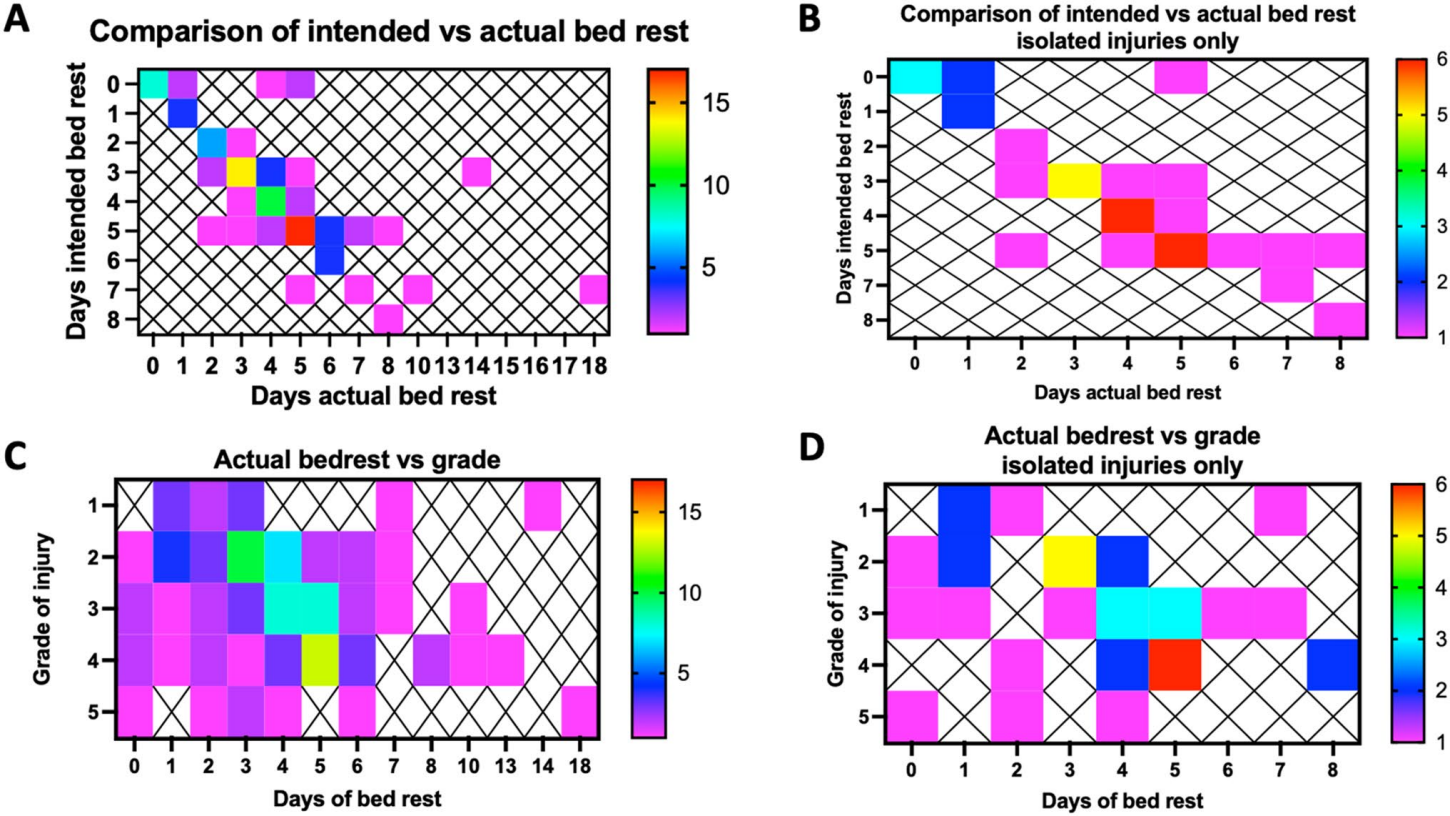
**A** and **B** Comparison between the number of days of intended bed rest and the days of bed rest actually given for all patients and those with only liver or spleen injury. **C** and **D** Comparison between the grade of injury and the number of days of bed rest given for all patients and those with only liver or spleen injury. The frequency of patients undergoing the specified duration of bedrest is demonstrated using the colour bar. A low frequency is demonstrated with purple and dark blue colours, a moderate frequency with light blue and green colours and a high frequency with yellow and red colours

Bed rest was employed in 114/126 (90%) of children in whom location was reported on the first day of admission, despite only 34/126 (27%) being admitted to ICU (Table 2). By day 5 after injury only 5/73 (7%) patients who remained in hospital were cared for in ICU but 42/73 (58%) remained on bed rest (Fig. 1). A significant variation in practice around bed rest is evident, even in patients with isolated injury (Fig. 1D). Four patients on the ward were tachycardic on day 5, two of whom required a fluid bolus, demonstrating that a small number of children who are potentially unstable may be cared for in a ward rather than ICU setting.

**Table 2:** Association between location of care, use of bed rest and median grade of injury evaluated using AAST grading

Days after injury	Location of care	Bed rest	Median injury grade (IQR)
1	ED—5	5/5 (100%)	3 (2; 3)
	ICU—34	33/34 (97%)	3 (2; 4)
	HDU—22	21/22 (95%)	4 (3; 4)
	Ward—64	55/64 (86%)	3 (2; 3.5)
	Not recorded—5		
2	ICU—24	24/24 (100%)	3 (2; 4)
	HDU—12	12/12 (100%)	4 (2.5; 4)
	Ward—81	64/81 (79%)	3 (2; 4)
3	ICU—15	14/15 (93%)	3 (2; 4)
	HDU—6	4/6 (67%)	4 (3.3; 4.8)
	Ward—80	63/80 (79%)	3 (2; 4)
4	ICU—8	5/8 (63%)	3 (2; 4)
	HDU—5	4/5 (80%)	3.5 (3; 4.3)
	Ward—74	53/74 (72%)	3 (2; 4)
5	ICU—5	5/5 (100%)	3 (2; 4)
	HDU—2	2/2 (100%)	3.5 (3.3; 3.8)
	Ward—66	35/66 (53%)	3 (2; 4)

*ED* Emergency Department, *ICU* Intensive Care Unit, *HDU* High Dependency Unit

### Survey respondents

62 (63%) respondents use radiological grade of injury all of the time to determine the duration of prescribed bed rest after BLSI. 16 (16%) use the grade of injury some of the time to determine the duration of bed rest and most commonly these are injuries grade III or above (8/16, 50%) or grade IV or above (5/16, 31%) but 3/16 (19%) only used it for low grades of injury. 2 (2%) respondents use the same duration of best rest for all patients. The remaining 19 (19%) respondents use a combination of haemodynamic status (most commonly), pain, age, Haemoglobin level, other injuries and imaging findings (other than grade) to determine the duration of bed rest. No respondents report using location of care (i.e. ICU vs ward) as a determinant of bed rest.

**APSA guidance: discharge should be based on clinical condition, not injury severity. This includes haemodynamic stability, minimal abdominal pain and tolerating diet.**

### Level III–IV evidence, grade C recommendation [5]

Table 3 displays the criteria that were documented in children’s records as forming a decision in their readiness for discharge and those which respondents use to determine readiness for discharge. Grade of injury and repeat imaging were frequently reported to be an important determinant of readiness for discharge.

**Table 3:** Criteria used for discharge after Blunt Liver and Spleen Injury

Criteria	Patients		
	isolated liver or spleen or both only (*n* = 54)	Multiple injuries (*n* = 77)	Survey respondents *N* (%)
Grade of injury plus one day only	0	0	6 (6%)
Grade of injury plus one day in combination with other features	19 (35%)	38 (49%)	61 (62%)
Observations normal	39 (72%)	63 (82%)	89 (90%)
Tolerating diet	41 (76%)	62 (81%)	82 (83%)
Stable haemoglobin	28 (52%)	38 (49%)	79 (80%)
Pain free	35 (65%)	45 (58%)	67 (68%)
Resolving injury or absence of pseudoaneurysm on imaging	14 (26%)	16 (21%)	16 (16%)

**APSA guidance: restricting activity to grade plus 2 weeks is safe. Shorter restrictions may be safe but there is inadequate data to support decreasing these recommendations.**

### Level III–IV evidence, grade C recommendation [5]

25/99 (25%) of survey respondents report using grade of injury plus 2 weeks to determine the duration of restricted activity, although this advice was only given to 12/131 (9%) of patients. 28/99 (28%) reported restricting activity for 6 weeks for all patients and this advice was given to 34/131 (26%) of patients. 22/99 (22%) survey respondents report restricting activity until they have reviewed them as an outpatient and this advice was given to 7/131 (5%) of patients. 55 (42%) patients did not have any documented advice about return to activity. The remaining 23 patients received a variety of advice ranging between limiting activity for between 2 weeks and 3 months, with a restriction due to concomitant injury in 5 patients.

**APSA guidance: consider imaging for symptomatic patients with prior high grade injuries.**

### Level IV evidence, grade C recommendation [5]

Table 4 displays the reported approach to routine imaging (subsequent to the initial CT) after BLSI, describing the timing and modality that is used. Respondents could select more than one response if they routinely image more than once. Some respondents noted that they specifically look for pseudoaneurysm prior to discharge.

**Table 4:** Reported use of routine imaging after BLSI

Timing /criteria for follow-up imaging (*n* = 99)	Modality of imaging used (*n* = 99)				
	Spleen	Liver	Modality	Spleen	Liver
No routine imaging	41 (41)	38 (38)	USS	53 (54)	51 (52)
Within 1 week	28 (28)	32 (32)	CE-USS	8 (8)	9 (9)
Within 1 month	8 (8)	12 (12)	CT	5 (5)	6 (7)
Within 3 months	26 (26)	25 (25)	MRI	0 (0)	2 (2)
High grade/hilar injuries only	6 (6)	6 (6)	HIDA	N/A	3 (3)

Data are number (%) of respondents

*USS* ultrasound scan, *CE-USS* contrast enhanced ultrasound scan, *CT* computed tomography, *MRI* magnetic resonance imaging, *HIDA* hepatobiliary iminodiacetic acid scan

56/130 (43%) patients had further imaging after their initial CT to reassess their liver or spleen injury before discharge from hospital. The grade of injury was significantly higher in those who had any re-imaging before discharge compared to those who did not (3 (IQR 2–4) vs 2.5 (IQR 2–4), p 0.02). At the time of the first (and in many, only) re-imaging, 28 (50%) patients were asymptomatic, 15 (27%) had mild abdominal pain without other symptoms, 9 (16%) had moderate abdominal pain, some with additional symptoms and 2 (4%) had severe pain and/or additional symptoms at the time of imaging. Overall 41 doppler ultrasound scans (d-USS), 8 contrast enhanced ultrasound scans (CE-USS), 21 CT scans and 4 other scans (HIDA or MRI) were used to re-assess the liver or spleen before discharge. One pseudoaneurysm was detected and treated by interventional radiology in a mildly symptomatic patient with a grade II injury. After discharge 43 (33%) patients underwent further imaging of their liver or spleen—d-USS was performed in 37 and CT or CE-USS in the other 5. Overall, 74 (56%) had a least one further radiological assessment of their liver or spleen injury after the initial diagnostic CT.

## Discussion

In this report we demonstrate that there is variability in the intended and given care for children with blunt liver and spleen injury (BLSI) in UK paediatric major trauma centres. Current UK practice deviates from recently published guidance from APSA in key areas of management including duration of bed rest, criteria for discharge and follow-up imaging.

There is now a strong evidence base for basing the location of care on haemodynamic stability rather than grade of injury [5, 10] and this is reflected in both the survey and prospective data collection. Clinicians reported that their main criteria for ICU admission was haemodynamic stability alone or in combination with other factors, rather than grade of injury alone. This approach was generally reflected in patient management, with almost 2/3 of those admitted to ICU requiring fluid resuscitation and 70% having multiple injuries. A small number of patients were admitted to ICU without either of these indications, suggesting that there is still scope to improve ICU utilisation further in this group of patients.

APSA guidance recommends bed-rest on ICU until vital signs are normal, then no further bed rest. All but one respondent report using bed-rest within their management plan after BLSI and for the majority, the duration was determined by radiological grade of injury. Haemodynamic stability was the main factor for bed-rest duration in those respondents not using grade and this may be a more appropriate determinant for mobilisation, given that there are many factors which affect whether a child is admitted to ICU. The use of therapeutic bed rest within clinical practice is prevalent, with almost 60% of those in hospital on day 5 after injury remaining on bed rest in a ward setting. The reasons for this may be multi-factorial: thresholds for step-down from ICU may differ in the UK compared to the USA, where the majority of studies have been undertaken; the decision to moblise may be determined by patients’ symptoms; and it may be impacted by their concomitant injuries. Whilst there is a growing evidence base for early mobilisation after blunt liver and spleen injury [21–23], studies have not followed the protocol recommended by the APSA guidance.

The reported and observed approach to imaging after initial CT differs significantly from the APSA guidance which notes that delayed splenic bleeding has a low reported incidence of 0.2–0.3% [24, 25] and that complications rarely arise in asymptomatic individuals [5]. Despite this, over 50% of respondents report that they routinely image patients after injury and a similarly high proportion of patients underwent further imaging. This may be because the rate of pseudoaneurysm after BLSI is reported to be as high as 22% [5, 26], because pseudoaneurysms have been detected after grade II splenic injuries [27] and patients have suddenly decompensated with bleeding 10 days after injury, having previously being well [26, 28]. Respondents reported that they image at two main times—within the first week and within 3 months of injury—suggesting that complications are looked for prior to discharge from hospital and before discharge from follow-up.

There was no clear approach from respondents about the advice given for returning to normal activity, with practice varying and a surprisingly high proportion of patients not receiving documented advice about this. A recent article showed that when using the APSA guidelines of grade of injury + 2 weeks to return to sport in a cohort of 1005 patients there were no episodes of re-bleeding, whether patients followed the guidance or not [29]. Knowledge and experience of significant complications, particularly delayed bleeding after trauma, may be major factors for clinicians not following APSA guidance. Further systematic research is needed to determine a safe, stratified approach to the modality and timing of imaging. Furthermore, developing evidence to guide standardised advice for safe return to activity and contact sports is important for many children with BLSI, some of whom play sport competitively [30].

Adherence to trauma guidance in general has been shown to be variable and in some cases based on the strength of the evidence behind the recommendations [31]. Proven evidence based best-practice after trauma have also been demonstrated to occur more frequently in larger hospitals and level I trauma centres [32]. Mortality rates after paediatric blunt abdominal trauma are very low, as demonstrated in the results of this study, making mortality a poor indicator of high quality care on which to determine the impact of the guidance. A core outcome set (COS) has been developed for the evaluation of outcomes after damage control laparotomy [33] but a COS does not exist for children (or adults) managed conservatively for abdominal trauma. The results of this paper highlight key areas where future research should be focussed to advance the evidence base and therefore guidance. A COS as a first step towards this would determine the important outcomes to be reported in publications regarding blunt abdominal trauma.

### Limitations and strengths

This study describes practice from all of the Paediatric Major Trauma Centres in the UK with a good individual response rate of over 60%, giving a robust national overview of specialist practice. The nature of a survey such as this may oversimplify the management of complex injuries and the nuance of individual patient situations which is recognised as a limitation. The number of expected patients has been achieved, demonstrating an excellent uptake of the audit enabling it to be representative of national practice in pMTCs. A significant proportion of the patients in this series have concomitant injuries making some conclusions difficult to draw, particularly around criteria for admission to ICU and duration of bed rest.

## Conclusions

The management of paediatric BLSI in the UK deviates from the APSA guidance in many areas. There is common ground on the indications for admission to ICU and the use of clinical features to determine readiness for discharge, demonstrating strong support for this aspect of the guidance with further scope to improve adherence to this aspect of the guidance in UK practice.

UK surgeons continue to place significant emphasis on radiological grade of injury to determine duration of bed rest yet there is a good evidence base that an abbreviated bed rest duration is safe. The majority of UK specialists routinely re-image children after BLSI using ultrasound scan and guidance about return to activity is variable. These deviations from the guidance occur where the evidence base is weak, highlighting that further robust research focussing on the natural history of pseudoaneurysm and return to activity is warranted.

### Supplementary Information

Below is the link to the electronic supplementary material.

Supplementary file1 (DOCX 35 KB)

Supplementary file2 (DOCX 22 KB)
