# Supplementary material for: The Blunt Liver and Spleen Trauma (BLAST) audit: national survey and prospective audit of children with blunt liver and spleen trauma in major trauma centres
Source: Eur J Trauma Emerg Surg. 2022 Jun 21;49(5):2249–56. doi: 10.1007/s00068-022-01990-3 (PMC10520113; doi:10.1007/s00068-022-01990-3)
Supplement: Supplementary file 2 — Supplementary file2 (DOCX 22 KB) [file 68_2022_1990_MOESM2_ESM.docx]

**Supplementary Information**

CHERRIES checklist for e-surveys

| Title | Description | Location in manuscript or description |
| --- | --- | --- |
| Design | Describe survey design | Page 6 |
| IRB approval and informed consent process | IRB approval | Page 8 |
|  | Informed consent | Page 8 |
|  | Data protection | No identifiable information was held |
| Development and pre-testing | Development and pre-testing | Page 7 |
| Recruitment process and description of the sample with access to the questionnaire | Open survey vs closed survey | Page 7 |
|  | Contact mode | Page 7 |
|  | Advertising the survey | n/a |
| Survey administration | Distribution | Page 7 |
|  | Context | n/a |
|  | Mandatory/voluntary | Voluntary |
|  | Incentives | n/a |
|  | Time/date | Page 7 |
|  | Randomisation | n/a |
|  | Adaptive questioning | Not used |
|  | Number of items | Supplementary information |
|  | Number of screens | Page 7 |
|  | Completeness check | Yes |
|  | Review step | No |
| Response Rate | Completion Rate | Page 9 |
| Preventing multiple entries |  | Page 9 |
| Analysis | Handling of incomplete questionnaires | Page 7 |

**Survey Questions**

1. Which hospital do you mainly practice in?

2. What age of child with blunt liver or spleen injury do you care for? (select

all that apply)

a. <12 years

b. >= 12 years

c. <16 years

d. <18 years

e. Other (free text)

3. Do you have a special interest/sub-specialist area?

a. No

b. Thoracic

c. Upper GI

d. Lower GI

e. Urology

f. Oncology

g. Neonatology

h. Trauma

i. Hepato-biliary

4. Do you have 24 hours access to interventional radiology on site?

a. Yes

b. No

5. Do you routinely place certain children with isolated liver or spleen injury

in HDU?

Yes/No

6. If yes, if there a specific grade of injury that you would base this decision

on?

a. Grade I

b. Grade II

c. Grade III

d. Grade IV

e. Grade V

f. No

g. Not based on grade (elaborate, free text)

7. Do you routinely place certain children with isolated liver or spleen injury

in PICU?

Yes/No

8. If yes, if there a specific grade of injury that you would base this decision

on?

a. Grade I

b. Grade II

c. Grade III

d. Grade IV

e. Grade V

f. No

g. Not based on grade (elaborate, free text)

9. If there are no complicating factors, the patient is haemodynamically

stable and has not required a transfusion do you use their radiological

grade of injury to determine the duration of bed rest?

a. No

b. Yes – for all grades of injury

c. Yes – for some grades of injury

d. Other, please describe (free text)

10. Which of the following criteria do you use for discharge (can select more

than one)

a. Pain free

b. Hb stable

c. Vitals normal

d. Tolerating diet

e. Completed bedrest of grade of injury + 1 days

f. Resolving injury on imaging

g. Other (free text)

11. When do you advise children to return to sport after injury?

a. Grade of injury + 2 weeks

b. After 6 weeks

c. After routine review in outpatient clinic

d. Other (free text)

12. Do you routinely image children after spleen injury has been confirmed

by Computerised Tomography? (can select more than one)

a. Yes – within the first week after injury

b. Yes – within the first month after injury

c. Yes – within the first 3 months after injury

d. Other (free text)

13. If you do routinely image children after splenic injury, which modality do

you use? (can select more than one)

a. Ultrasound scan

b. Computerised Tomography

c. Magnetic Resonance Imaging

d. Other (free text)

14. Do you routinely image children after liver injury has been confirmed by

Computerised Tomography? (can select more than one)

a. Yes – within the first week after injury

b. Yes – within the first month after injury

c. Yes – within the first 3 months after injury

d. Other (free text)

15. If you do routinely image children after liver injury, which modality do

you use? (can select more than one)

a. Ultrasound scan

b. Contrast enhanced ultrasound scan

c. HIDA scan

d. Computerised Tomography

e. Magnetic Resonance Imaging

f. Other (free text)
